# Prevalence and complications of nonsurgical hypoparathyroidism in Korea: A nationwide cohort study

**DOI:** 10.1371/journal.pone.0232842

**Published:** 2020-05-08

**Authors:** Se Hwa Kim, Yumie Rhee, Yoo Mee Kim, Young Jun Won, Junghyun Noh, Hyemi Moon, Juneyoung Lee, Sin Gon Kim

**Affiliations:** 1 Department of Internal Medicine, International St. Mary’s Hospital, Catholic Kwandong University College of Medicine, Incheon, Republic of Korea; 2 Department of Internal Medicine, Yonsei University College of Medicine, Seoul, Republic of Korea; 3 Department of Internal Medicine, Inje University Ilsan Paik Hospital, Goyang, Republic of Korea; 4 Department of Biostatistics, Korea University College of Medicine, Seoul, Republic of Korea; 5 Department of Internal Medicine, Korea University College of Medicine, Seoul, Republic of Korea; University of Hull, UNITED KINGDOM

## Abstract

**Objective:**

The risk of complications of nonsurgical hypoparathyroidism in Asia is unclear. We estimated the prevalence and risk of complications in patients with nonsurgical hypoparathyroidism.

**Methods:**

We performed a retrospective cohort study using a nationwide claims database from 2005 to 2016. Among the entire Korean population, we identified 897 patients diagnosed with nonsurgical hypoparathyroidism during 2005–2015. We selected 210 patients with nonsurgical hypoparathyroidism during 2005–2008 who had no complications at baseline and followed them to 2016. Control subjects (n = 2075) were matched using propensity scores based on age, sex, and comorbid disease with a 1:10 ratio and monitored until 2016.

**Results:**

The age-standardized prevalence of nonsurgical hypoparathyroidism was 0.2 cases per 100,000 persons in 2005. During a mean follow-up period of 9.5 years, patients with nonsurgical hypoparathyroidism had a higher risk of cardiovascular disease, especially arrhythmia (hazard ratio [HR], 2.03; 95% confidence interval [CI], 1.11–3.70) and heart failure (HR, 2.43; 95% CI, 1.22–4.83). The risk of vertebral fracture was higher in patients than in controls (HR, 2.27; 95% CI, 1.09–4.72). Patients had a significantly increased risk of renal disease (HR, 2.57; 95% CI, 1.56–4.21), seizure (HR, 5.74; 95% CI, 3.34–9.86), depression and bipolar disease (HR, 1.82; 95% CI, 1.30–2.56), and cataract (HR, 1.90; 95% CI, 1.30–2.79) compared with controls.

**Conclusions:**

The prevalence of nonsurgical hypoparathyroidism was very low in Korea but was associated with a higher risk of incident cardiovascular disease and vertebral fracture as well as known complications including renal disease, seizure, and cataract.

## Introduction

Hypoparathyroidism is a rare disease characterized by hypocalcemia with inappropriately low parathyroid hormone (PTH) levels. Hypoparathyroidism can be divided into primary hypoparathyroidism due to intrinsic defects of the parathyroid glands, including genetic causes, and a secondary form due to several causes including neck surgery, autoimmune disease, or other rare infiltrative diseases [1]. Neck surgery is the most common cause of hypoparathyroidism (postsurgical hypoparathyroidism), accounting for approximately 75% of cases. Autoimmune hypoparathyroidism is the next most common form in adults and may be isolated or part of an autoimmune polyglandular syndrome. Worldwide, there are few reports on the prevalence of nonsurgical hypoparathyroidism. The reported estimated prevalence has varied among studies and is reported to be 0.7/100,000 persons in Japan and 2.3/100,000 persons in Denmark [2,3]. Another study reported a prevalence of 4.8/100,000 persons in the United States [4].

Although the U.S. Food and Drug Administration approved recombinant human PTH (1–84) for managing hypoparathyroidism, high-dose calcium and active vitamin D has been the main hypoparathyroidism treatment in most countries, generating concerns for unwanted complications of hypercalciuria, renal stones, impaired renal function, and cardiovascular disease (CVD) [1].

To date, there has been no report on the risk of complications of nonsurgical hypoparathyroidism in Asia. Herein, we estimated the crude prevalence of nonsurgical hypoparathyroidism in Korea using a large claims database. We also assessed the risks of complications and mortality of patients with nonsurgical hypoparathyroidism compared with age-, sex-, and comorbidity-matched controls. To the best of our knowledge, this is the first matched cohort study using a nationwide database performed in Asia.

## Materials and methods

### Data source and study population

The National Health Information Database (NHID) is a public database on healthcare utilization, health screening, sociodemographic variables, and mortality for the entire population of South Korea, formed by the National Health Insurance Service [5]. The NHID contains prescription drugs and treatment claims records for nearly all Koreans. From among 50 million people in the NHID, we identified patients with nonsurgical hypoparathyroidism from 2005 through 2016. First, we identified patients with nonsurgical hypoparathyroidism based on the International Classification of Diseases 10^th^ revision (ICD 10) codes (D82.1, E20.0, E20.8, E20.9, E31.0, E31.8, and E31.9) and at least two prescriptions for active vitamin D analogs. Next, we excluded subjects who had a history of head and neck cancer, thyroid or parathyroid surgery, radiation to the neck region, or stage 5 chronic kidney disease during 2002–2016. We further excluded subjects who had renal insufficiency within 3 years before and within 1 year after the index date to rule out secondary hypocalcemia due to renal insufficiency. We also excluded infants who were followed up <1 year after the index date to exclude neonatal transient hypoparathyroidism.

To examine the risks of complications and mortality of nonsurgical hypoparathyroidism, we compared the prevalence of complications and mortality in a nonsurgical hypoparathyroidism group and a control group. First, as case subjects, we selected 392 patients with newly diagnosed nonsurgical hypoparathyroidism during 2005–2008. After excluding 155 patients who had complications within 3 years before the index date and 26 patients who were followed up for <12 months, 211 patients with nonsurgical hypoparathyroidism were selected as cases. The control population consisted of participants who did not have ICD codes of nonsurgical hypoparathyroidism or any prescription of active vitamin D analogs. The exclusion criteria for controls were the same as for cases. Control subjects were then selected from the control population using 1:10 propensity score matching with the cases, which consisted of 2,075 subjects. Variables for constructing the propensity scores were age, sex, and comorbid disease of either diabetes or hypertension. Dyslipidemia was not used as a matching variable owing to its very low frequency; however, it was adjusted for use in a subsequent multivariable model. All subjects were followed up until December 31, 2016 using the NHID or until the subject’s death, which was ascertained from the Resident Register of Korea [6]. This study was approved by the Institutional Review Board of the Catholic Kwandong University, Republic of Korea (IRB No. CKU-IRB050). As this study was based on the NHID, the Institutional Review Board did not require informed consent from individual subjects. Data were fully anonymized and de-identified for the analysis.

### Comorbid disease and complications of nonsurgical hypoparathyroidism

Comorbid disease included diabetes mellitus, hypertension, and dyslipidemia. The diagnoses of these diseases were determined according to ICD-10 codes and prescriptions of corresponding medications for each disease: diabetes (E10-E14), hypertension (I10-I15), and dyslipidemia (E78). Any diagnosis of a complication during 2006–2016 was considered an incident episode in both groups. Complications were selected using three- or four-digit ICD-10 codes as follows: ischemic heart disease (I20, I24, I25), acute myocardial infarction (I21-I23), arrhythmia (I44-I45, I47-I49), heart failure (I50), cerebral infarction (I61-I64, I67.9), renal stones (N20-N23), renal insufficiency (N18-N19), seizure (G40-G41), cataract (H25, H26, H28.1), depression and bipolar disease (F30-F34), fracture (S52.5, S52.6, S42.2, S42.3, S72.0, S72.1, S22.0, S22.1, S32.0, M48.4, M48.5), and intracranial calcification (G23.8)

### Statistical analysis

Baseline demographic and clinical characteristics of patients are summarized as median (minimum-maximum) or mean (SD) for continuous variables and as number (percent) for categorical variables. Prevalence was age-standardized to the 2010 Korean population based on the 2010 National Census of Statistics Korea and expressed per 100,000 persons. The 1:10 propensity scores for matching cases to controls were made using a caliper width of 0.2 of the SD of the logit of the propensity score. Considering matched data, a stratified multiple Cox proportional hazards regression analysis was performed to determine significant complications of nonsurgical hypoparathyroidism. Adjusted hazard ratios (HRs) and their 95% confidence intervals (CIs) were calculated.

All analyses were performed using SAS software version 9.4 (SAS Institute, Cary, NC) and STATA software version 15.0 (Stata Corp., College Station, TX), and a two-sided *P* < 0.05 was considered statistically significant.

## Results

### Prevalence, incidence, and clinical characteristics of nonsurgical hypoparathyroidism

A total of 897 patients with nonsurgical hypoparathyroidism were identified from 2005 through 2015. The age-standardized prevalence of nonsurgical hypoparathyroidism was 0.2 cases per 100,000 persons in 2005 and 1.1 cases per 100,000 persons in 2015. The crude incidence of nonsurgical hypoparathyroidism was 0.18 cases per 100,000 person-years in 2005 and 0.13 cases per 100,000 person-years in 2015. The clinical characteristics of the 897 patients with nonsurgical hypoparathyroidism are shown in Table 1. The mean (± standard devidation) age was 43.8 ± 21.9 years, and 59% of patients were female. Four percent of patients were diagnosed with DiGeorge syndrome. Among 897 patients, 504 (56%) had no complications of nonsurgical hypoparathyroidism at the time of their diagnosis. The prevalence of seizure, cataract, depression and bipolar disease, and CVD at baseline was more than 10% in all patients.

**Table 1:** Clinical characteristics of all patients with nonsurgical hypoparathyroidism from 2005 through 2015 (n = 897).

Variable	Number (% or range)^a^
Female sex	529 (59%)
Age at baseline, years, mean (SD)	43.8 (21.9)
Diagnosis (code), n (%)	
DiGeorge syndrome (D82.1)	36 (4.0%)
Autoimmune polyglandular failure (E31.0)	1 (0.1%)
Idiopathic or unspecified hypoparathyroidism (E20.0, E20.8, E20.9)	860 (95.9%)
Medication, median dose (min-max)	
Vitamin D analogs, μg/day	
Calcitriol only (n = 482)	0.25 (0.25–1.0)
Alfacalcidol only (n = 259)	0.5 (0.5–2.0)
Calcifediol only (n = 6)	20 (20–20)
Mixed (n = 150)	NE
Calcium supplements	
Elemental dose, mg/day	207 (100–1487)
Complications at baseline, n (%)	393 (43.8%)
Cardiovascular disease	139 (15.5%)
Renal stones	9 (1.0%)
Seizures	148 (16.5%)
Cataract	100 (11.2%)
Depression and bipolar disease	125 (13.9%)
Fracture	25 (2.8%)
Intracranial calcification	4 (0.5%)

NE, not evaluable.

### Complications and mortality of nonsurgical hypoparathyroidism

After propensity score matching, the baseline characteristics of both groups were well balanced (Table 2). None of the subjects had any complications at baseline. During a median follow-up period of 9.5 years, the prevalence of any complication was higher in the nonsurgical hypoparathyroidism cases than in the controls (52.4% vs. 30.3%; HR, 2.14; 95% CI, 1.74–2.62; *P* < 0.0001) (Table 3). However, the risk of mortality in the case group was not significantly increased compared with that in the control group (HR, 1.56; 95% CI, 0.86–2.84; *P* = 0.145)

**Table 2:** Baseline characteristics of patients with nonsurgical hypoparathyroidism and control group.

Variables	Cases (n = 210)	Controls (n = 2,075)	*P*-value^a^
Age, mean (SD)	39.2 (19.9)	39.2 (19.9)	0.408
Female sex	131 (62.4)	1,299 (62.6)	0.813
Case enrollment year			
2005	49 (23.3)	465 (22.4)	
2006	59 (28.1)	590 (28.4)	
2007	59 (28.1)	590 (28.4)	
2008	43 (20.5)	430 (20.7)	
Comorbidities			
Diabetes	5 (2.4)	30 (1.5)	0.424
Hypertension	34 (16.2)	289 (13.9)	0.284
Dyslipidemia	3 (1.4)	5 (0.2)	NE

NE, not estimated due to low frequency.

Values given are number (%) unless indicated otherwise.

^a^*P*-values were obtained using a generalized estimating equation for matched data.

**Table 3:** Complications following nonsurgical hypoparathyroidism.

	Cases (n = 210)	Controls (n = 2,075)	aHR (95% CI)	*P*-value
Any complications	110 (52.4)	629 (30.3)	2.14 (1.74–2.62)	<0.0001
CVD				
IHD	14 (6.7)	126 (6.1)	1.04 (0.60–1.79)	0.902
AMI	2 (1.0)	10 (0.5)	2.03 (0.41–9.93)	0.384
Arrhythmia	13 (6.2)	63 (3.0)	2.03 (1.11–3.70)	0.021
Heart failure	9 (4.3)	38 (1.8)	2.43 (1.22–4.83)	0.012
Cerebral infarction	11 (5.2)	77 (3.7)	1.47 (0.79–2.73)	0.223
Any CVD	39 (18.6)	171 (8.2)	1.64 (1.20–2.26)	0.002
Renal complications				
Renal stones	10 (4.8)	46 (2.2)	2.13 (1.10–4.13)	0.025
Renal insufficiency	9 (4.3)	27 (1.3)	3.44 (1.63–7.23)	0.001
Any renal complication	18 (8.6)	71 (3.4)	2.57 (1.56–4.21)	0.0002
Seizures	17 (8.1)	31 (1.5)	5.74 (3.34–9.86)	<0.0001
Cataract	32 (15.2)	179 (8.6)	1.90 (1.30–2.79)	0.001
Depression and bipolar disease	44 (21.0)	258 (12.4)	1.82 (1.30–2.56)	0.0006
Fracture				
Humerus or wrist fracture	7 (3.3)	71 (3.4)	0.92 (0.42–2.01)	0.836
Vertebral fracture	9 (4.3)	39 (1.9)	2.27 (1.09–4.72)	0.029
Hip fracture	0 (0.0)	11 (0.5)	NE	
Any fracture	15 (7.1)	116 (5.6)	1.26 (0.73–2.16)	0.404
Intracranial calcification	2 (1.0)	0 (0.0)	NE	
Death	13 (6.2)	81 (3.9)	1.56 (0.86–2.84)	0.145

aHR, adjusted hazard ratio; AMI, acute myocardial infarction; CI, confidence interval; CVD, cardiovascular disease; IHD, ischemic heart disease; NE, not evaluable.

Values given are number (%) unless indicated otherwise. The aHR (95% CI) and *P*-values were obtained using a stratified Cox proportional hazards regression analysis.

#### Cardiovascular disease

**Fig 1:**
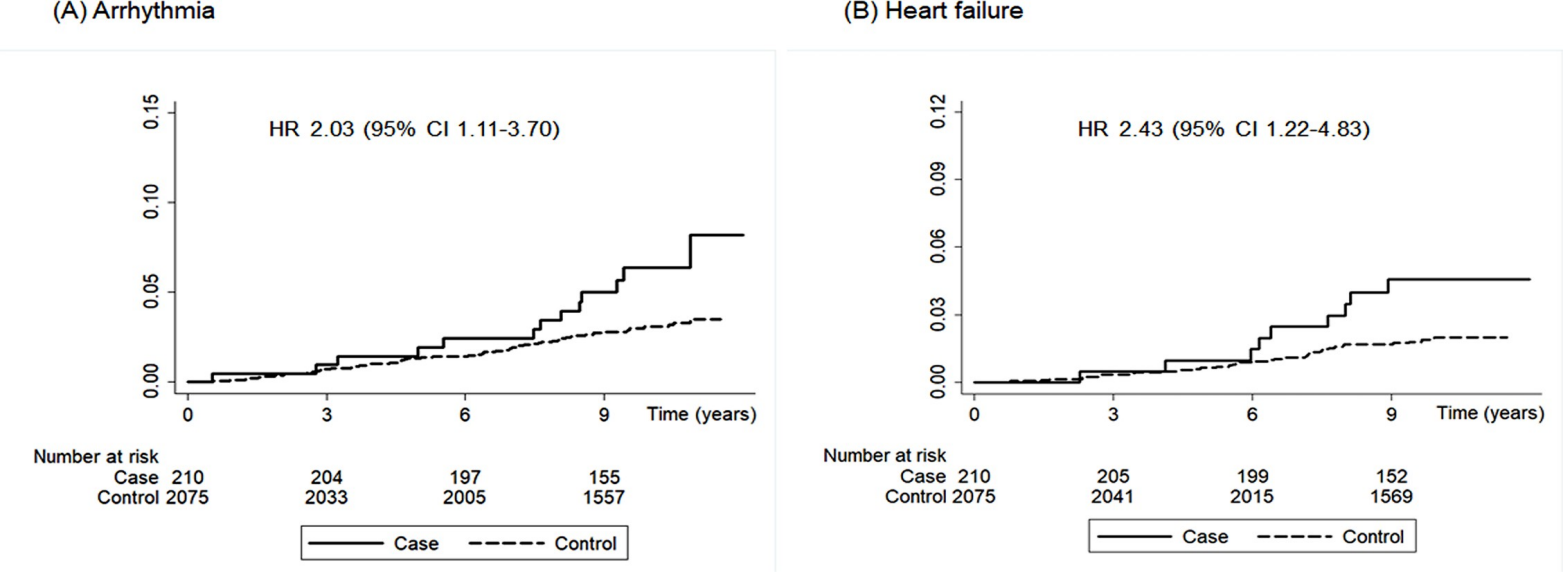
Kaplan-Meier survival curve of arrhythmia (A) and heart failure (B) in patients with nonsurgical hypoparathyroidism and controls. Hazard ratios (HRs) with 95% confidence intervals (CIs) are shown.

Overall, the risk of any CVD event was significantly increased in patients with nonsurgical hypoparathyroidism compared with controls during the follow-up period (HR, 1.64; 95% CI, 1.20–2.26, *P* = 0.002) (Table 3). This was because of a significantly increased risk of heart failure (HR, 2.43; 95% CI, 1.22–4.83; *P* = 0.012) and arrhythmia (HR, 2.03; 95% CI, 1.11–3.70; *P* = 0.021) in the patient group (Fig 1). The risk of ischemic heart disease and cerebral infarction did not differ between the groups.

#### Fractures

There was no difference in the prevalence of any type of fracture between the groups (7.1% for cases vs. 5.6% for controls, *P* = 0.404). However, the risk of vertebral fracture was higher in the case group than in the control group (HR, 2.27; 95% CI, 1.09–4.72; *P* = 0.029) (Table 3).

#### Other complications

The risk of any renal complication was higher in the case group than in the control group (HR, 2.57; 95% CI, 1.56–4.21; *P* = 0.0002) (Table 3). Specifically, the risk of renal insufficiency was approximately three times higher in patients than in controls (HR, 3.44; 95% CI, 1.63–7.23; *P* = 0.001) and the risk of renal stones was higher in the case group (HR, 2.13; 95% CI, 1.10–4.13; *P* = 0.025). Patients with nonsurgical hypoparathyroidism had a higher risk of seizure than did controls (HR, 5.74; 95% CI, 3.34–9.86; *P* < 0.0001). The prevalence of cataract was significantly higher in the case group than in the control group (HR, 1.90; 95% CI, 1.30–2.79; *P* = 0.001). Furthermore, the case group had a higher prevalence of depression and bipolar disease than did controls (HR, 1.82; 95% CI; 1.30–2.56; *P* = 0.0006).

## Discussion

### Principal findings

Our study represents a large-scale assessment of nonsurgical hypoparathyroidism using a well-documented nationwide database. In this propensity-weighted cohort study, patients with nonsurgical hypoparathyroidism had an increased risk for CVD and vertebral fracture as well as known complications of hypoparathyroidism such as renal disease compared with controls. Regarding CVD, the risk of arrhythmia and heart failure was significantly increased in patients with nonsurgical hypoparathyroidism.

### Interpretation and differences in relation to other studies

Worldwide, reports on the prevalence of nonsurgical hypoparathyroidism are rare. Nakamura et al. reported that the total number of patients with nonsurgical hypoparathyroidism was 900, for an estimated prevalence of 0.7/100,000 persons, in Japan [2]. Recently, Underbjerg et al. assessed the prevalence and complication risk of nonsurgical hypoparathyroidism using data from the Danish National Patient Registry and estimated its prevalence to be 2.3/100,000 persons [3]. We noted that the prevalence of age-standardized nonsurgical hypoparathyroidism was 1.1/100,000 persons in Korea.

Hypocalcemia can cause QT prolongation and induce arrhythmias [7]. Dilated cardiomyopathy and heart failure are known but rare complications of chronic hypocalcemia in patients with hypoparathyroidism, as described in a few case reports [8–11]. To date, few longitudinal cohort studies have investigated the risk of CVD including heart failure in patients with hypoparathyroidism. Our study found that the risk of heart failure was 2.4-fold higher in patients with hypoparathyroidism than in controls during 9.5 years of follow-up (4.3% vs. 1.8%). It was previously reported that the prevalence of heart failure in the general population was 1.5% in Korea, indicating that our result regarding the prevalence of heart failure is reliable [12]. Some plausible mechanisms exist to explain the increased risk of heart failure in patients with hypoparathyroidism. Calcium is an essential component for cardiac muscle contraction, and hypocalcemia has been shown to reduce cardiac contractility [13]. In hypoparathyroidism, serum calcium levels are preferentially maintained in the low-normal range or slightly below the normal range. Therefore, some patients treated with calcium and active vitamin D may have mild chronic hypocalcemia, which contributes to the increased risk of heart failure. It was also reported that vitamin D and PTH may have independent roles in cardiac function. Many studies have shown that there was a strong association between vitamin D deficiency and heart failure. Furthermore, vitamin D receptor knockout mice showed deterioration of heart function and accelerated myocardial remodeling [14,15]. It has been shown that PTH acts on voltage-gated calcium channels and exerts a positive chronotropic effect in neonatal cardiomyocytes [16]. In contrast, Mitchell et al. reported that one patient (0.8%) of 120 patients with hypoparathyroidism had dilated cardiomyopathy during 7.4 years of follow-up [17]. In another Danish cohort study, patients with nonsurgical hypoparathyroidism had an increased risk of CVD due mainly to ischemic heart disease and arrhythmia, but heart failure was not examined in this study [3].

Patients with chronic hypoparathyroidism have reduced bone remodeling, resulting in higher bone mass than age- and sex-matched controls [17–19]. However, few studies have reported a fracture risk in chronic hypoparathyroidism, and results have been inconsistent. Underbjerg et al. [3] found an increased risk of fracture of the forearm or proximal humerus (approximately 2.8-fold) in patients with nonsurgical hypoparathyroidism compared with controls, whereas the overall risk of fracture, including vertebral fracture, was similar between the hypoparathyroidism case and control groups. Our study did not show an increased risk of any fracture in patients with nonsurgical hypoparathyroidism, although the risk of vertebral fracture was two times higher in patients than in controls. Two small studies have investigated the risk of vertebral fracture in patients with postsurgical hypoparathyroidism. One study demonstrated that patients with hypoparathyroidism had more morphometric vertebral fractures than in age- and body mass index-matched controls [20], whereas another study found that patients with hypoparathyroidism had a lower incidence of spine deformity than controls [21]. Recently, Chawla et al. reported that patients with idiopathic hypoparathyroidism had a higher prevalence of morphometric vertebral fracture (odds ratio, 4.54; 95% CI, 1.28–16.04), although the bone mineral density of lumbar spine was significantly higher by 21.4% in patients with hypoparathyroidism than in controls [22]. Furthermore, they noted that the prevalence of vertebral fracture was greater in patients with hypoparathyroidism, especially in postmenopausal women and those on anticonvulsant therapy. Therefore, monitoring for vertebral fracture through lateral radiography of the spine and examination of clinical risk factors for fracture may be warranted in patients with nonsurgical hypoparathyroidism. To date, the long-term effects of low bone turnover and increased bone mass on fracture risk in chronic hypoparathyroidism remain unclear and need to be assessed further.

Generally, PTH stimulates renal tubular reabsorption of calcium. Therefore, hypoparathyroidism itself may increase the risk of hypercalciuria, and treatment with large doses of calcium supplements and active vitamin D can also increase the risk of hypercalciuria, renal stones, and renal insufficiency in hypoparathyroidism [1]. In our study, patients with nonsurgical hypoparathyroidism had a 2-fold increased risk of renal stone and a more than 3-fold increased risk of renal insufficiency compared with the general population (or control subjects). In the Danish cohort study, patients with nonsurgical hypoparathyroidism had a significantly increased risk of renal insufficiency (HR, 6.01; 95% CI, 2.45–14.75) but not an increased risk of nephrolithiasis (HR, 0.80; 95% CI, 0.17–3.85) [3].

Cataract is a frequent manifestation of chronic hypoparathyroidism. A low PTH level in hypoparathyroidism induces high phosphate levels, and treatment with high-dose calcium and active vitamin D may increase the calcium-phosphate product, which can induce calcium-phosphate crystal formation in the lens of the eyes [23]. In the Danish cohort study, patients with nonsurgical hypoparathyroidism had an increased risk of cataract (HR, 4.21; 95% CI, 2.13–8.34) compared with controls, similar to our result. However, another Danish study of postsurgical hypoparathyroidism showed no difference in the risk of cataract between patients and controls.

It is not clear whether nonsurgical hypoparathyroidism increases the risk of psychiatric disorder. In this study, the risk of depression and bipolar disease was increased in patients with nonsurgical hypoparathyroidism, which is consistent with findings from previous studies. One previous Danish case-control study found that neuropsychiatric complications were more prevalent in patients with nonsurgical hypoparathyroidism than in controls (HR, 2.45; 95% CI, 1.78–3.35). Furthermore, some studies showed an increased risk of depression and anxiety as well as an impaired quality of life in patients with hypoparathyroidism [24–26].

### Strengths and weaknesses

This study has several strengths and weaknesses. We used the NHID, which covers nearly the entire Korean population with a sample size of over 50 million. Therefore, our study captured nearly all patients with nonsurgical hypoparathyroidism in Korea and is the first large-scale study determining the prevalence and complications of nonsurgical hypoparathyroidism in Korea. The main limitation was that the information regarding the diagnosis of disease may not be optimal for identifying disease prevalence, as the data were not established for research purposes. Therefore, we added prescription data as a secondary criterion of diagnosis and used several exclusion variables to improve the accuracy of diagnosis. Additionally, because we did not perform medical chart reviews, we could not assess the exact dose of calcium supplements and active vitamin D analogs. Another limitation was the lack of biochemical data such as serum calcium levels at baseline and during the follow-up period. Finally, our findings were limited by potential residual and unrecognized confounding bias, even though we performed propensity score matching.

## Conclusion

Nonsurgical hypoparathyroidism is associated with an increased risk of heart failure and vertebral fracture as well as previously well-known complications such as renal stones and cataract. Careful screening is needed for long-term complications of nonsurgical hypoparathyroidism.
